# Identification of young adults at risk of an accelerated loss of kidney function in an area affected by Mesoamerican nephropathy

**DOI:** 10.1186/s12882-018-1193-x

**Published:** 2019-01-16

**Authors:** Marvin Gonzalez-Quiroz, Evangelia-Theano Smpokou, Neil Pearce, Ben Caplin, Dorothea Nitsch

**Affiliations:** 1 Research Centre on Health, Work and Environment (CISTA), National Autonomous University of Nicaragua at León (UNAN-León), Campus Médico, Facultad de Ciencias Médica, edificio C, León, Nicaragua; 2 Department of Non-Communicable Disease Epidemiology, London School of Hygiene and Tropical Medicine, London, UK; 3 Centre for Nephrology, University College London, London, UK

**Keywords:** Mesoamerican nephropathy, Chronic kidney disease of unknown aetiology, Prediction, Kidney function status, Serum creatinine, uNGAL, ROC, Nicaragua

## Abstract

**Background:**

After two-years of follow-up of 263 apparently healthy 18- to 30-year-old men in communities affected by Mesoamerican nephropathy (MeN), we identified three distinct case groups: a subgroup with (i) established renal dysfunction (case-group 1); individuals with (ii) a rapid decline in kidney function (case-group 2); and individuals with (iii) stable kidney function (non-cases). This paper investigates whether local tests are potentially useful for the timely identification of these case groups.

**Methods:**

Creatinine levels were measured in local laboratories every six months for two years. Aliquots were sent to a centralized laboratory for measurements of cystatin C and creatinine levels. We investigated agreement between the locally and centrally measured creatinine-based Chronic Kidney disease Epidemiology Collaboration (CKD-EPI) equation for estimating the Glomerular Filtration Rate (eGFR). A logistic regression analysis was used to assess predictive factors for case groups 1 and 2 compared to non-cases. Predictive variables were locally measured eGFR, and urinary neutrophil gelatinase-associated lipocalin (uNGAL) levels. The discrimination performance of the model was assessed using the area under the receiver operating characteristic curve (AUC).

**Results:**

Considerable variation in local eGFR measurements was observed. The prediction model for case-group 1 included baseline kidney function and with or without uNGAL (AUC = 0.98, 95% confidence interval (CI), 0.91–1.00). The prediction model for case-group 2 also required eGFR_Scr_ at six and twelve months after baseline, with or without uNGAL levels (AUC = 0.88; 95% CI 0.80–0.99).

**Conclusions:**

Established renal dysfunction was detected at a single time point using local measurements and uNGAL. For the detection of a rapid decline in kidney function over time, at least 2 more measurements at six and twelve months are needed.

**Electronic supplementary material:**

The online version of this article (10.1186/s12882-018-1193-x) contains supplementary material, which is available to authorized users.

## Background

Mesoamerican nephropathy (MeN) is a major public health and economic problem affecting rural and agricultural communities in Mesoamerica. Over the last decade, MeN, also known as chronic kidney disease of unknown aetiology (CKDu), has caused the deaths of thousands of vulnerable young male agricultural workers, particularly sugarcane workers and other workers (agricultural and non-agricultural) who work in extremely hot conditions along the Pacific coast of Mesoamerica [1–5]. MeN has devastating consequences for patients, family members, communities and the country, with patients who are diagnosed with the disease progressing to end stage renal disease as young and middle-aged adults. Renal replacement therapy options are expensive and limited in Mesoamerica, resulting in the high mortality of patients with MeN [6, 7]. 

MeN disease has some unique characteristics, including the absence of traditional risk factors (hypertension and diabetes) [1, 8, 9]. A recent systematic review identified that the male gender, living in lowlands, a family history of CKD and a high water-intake are associated with CKDu [10]. Some studies have reported elevated urinary neutrophil gelatinase-associated lipocalin (uNGAL) and N-acetyl-β-D-glucosaminidase (NAG) levels in sugarcane workers [11, 12]. Therefore, one current hypothesis is that MeN is caused by repetitive acute kidney injury due to multiple risk factors, such as working conditions and heat [1, 8].

We have recently conducted a community-based cohort study in the affected region. We used established biomarkers of kidney function (serum cystatin C (Scys) and serum creatinine (Scr) levels) that were measured in stored samples from baseline and 6-month follow-up visits in a central laboratory at the end of the study (five measures in total) to determine the decline in kidney function after 2 years of follow-up. [13, 14] Although we attempted to recruit people without established kidney disease at baseline, 25 people (10.5% of the cohort) who were apparently healthy but in fact had established kidney dysfunction at baseline were included. An additional sub-group of males (10.5% of the total cohort) exhibited an extremely rapid decline in kidney function of 18.2 mL/min/1.73 m^2^/year from normal levels [14]. The observed dramatic loss of renal function among a sizeable sub-group of an apparently healthy young population is a concern.

The local industries in Nicaragua have established baseline screening programmes for kidney dysfunction using a single local creatinine test [15]. Despite the use of this local screening programme, a sub-group of people with established kidney disease was recruited in our study; therefore, a question arises of whether other information is needed in addition to a single serum creatinine measurement to detect MeN. In addition, the identification of the subgroup that will experience a rapid decline in kidney function will be beneficial to provide advice (e.g., avoidance of known nephrotoxins, such as non-steroidal anti-inflammatory drugs) and potentially to implement interventions (e.g., improve work conditions, clean water etc., if these interventions are proven to be effective) to prevent or delay future kidney function loss. Serial measurements of serum creatinine levels over more than 2 years are not the usual practice in Nicaragua. Therefore, the purpose of this analysis is to determine if repeated local creatinine tests combined with urinary measurements of uNGAL levels can identify the subgroup of individuals at risk of a future rapid decline in kidney function.

## Methods

### Study population

These analyses are based on our existing community-based follow-up study of a decline in kidney function among affected communities in Nicaragua. The rationale and study design have been published elsewhere [13]. Briefly, we recruited 350 apparently healthy young adults aged 18–30 years without a known diagnosis of CKDu and traditional risk factors, and followed them for two years. All eligible healthy males and a sample of females from 9 communities in northwest Nicaragua were enrolled in our study. Biological samples, anthropometric measurements and questionnaire data were collected at baseline and then at six-month intervals. The outcome was the kidney function status, and we classified the participants into three categories: (i) established renal dysfunction; (ii) a rapid decline in kidney function; and (iii) stable kidney function [14].

### Clinical measurements

#### Gold standard

All samples were analysed in a single batch to reduce time-dependent measurement errors in the assays after two years of follow-up. At the end of the follow-up period, stored (− 80 °C) serum aliquots from all study visits were transferred to the Clinical Trial Service Unit at Oxford University. Scr levels were quantified using a Beckman AU680 Chemistry Analyser (Jaffe compensated method) and calibrated against the IDMS-traceable creatinine standard. Scys levels were measured using Siemens BN ProSpec (nephelometry) [14]. The CKD-EPI equation for serum creatinine and cystatin c levels was used to estimate the estimated glomerular filtration rate, or eGFR (eGFR_Scr-Scys_) [16].

#### Routine local measurements

Serum creatinine (Scr) levels were also measured locally (in the Biochemistry Department at the National Autonomous University of Nicaragua-Leon) using a ChemWell® 2910 (Awareness Technology, EEUU) auto analyser (Jaffe compensated method) [17, 18]. Local Scr values were multiplied by 0.95, as they were not calibrated to an IDMS-traceable creatinine standard [19]. Kidney function was calculated using the estimated glomerular filtration rate according to the CKD-EPI formula by determining Scr levels [16] at each study visit.

#### Urine markers

Creatinine and albumin levels in baseline urine samples, which were previously frozen and stored at − 80 °C for 2.5 years, were measured using the Jaffe and bromocresol green reactions (Sigma-Aldrich, MAK124), respectively. Samples were read at a specific wavelength using the Biochrom EZ Read 400 Microplate Reader. Urinary neutrophil gelatinase-associated lipocalin (uNGAL) levels were measured in a subsample. Samples from fifty-five randomly selected participants with stable kidney function and samples from all participants in the other two subgroups (rapid decline in kidney function and patients with established renal dysfunction; both of which are defined below) were analysed. uNGAL levels were measured using enzyme-linked immunoassay (ELH-Lipocalin2, RayBiotech), according to the manufacturer’s instructions. Albumin and uNGAL levels are reported as ratios to urinary creatinine levels measured using the Jaffe method (Sigma-Aldrich, C4255) [14].

### Definitions of outcome categories

A growth mixture model (GMM) was used to identify three sub-groups of kidney function in males using the gold standard eGFR after 2 years, as previously described [14]. A group with established renal dysfunction at baseline (mean eGFR_Scr-Scys_: 58 mL/min/1.73 m^2^) was investigated, which for the purpose of this paper, is defined as case-group 1. Then, a group with normal baseline kidney function (mean eGFR: 112 mL/min/1.73 m^2^) showed a rapid decline in eGFR_Scr-Scys_ of − 18.2 mL/min/1.73 m^2^/year and were designated case-group 2, i.e., rapid decliners. The remaining study participants were ‘non-cases’ with stable kidney function, i.e., men with persistently stable kidney function (mean baseline eGFR: 116 mL/min/1.73 m^2^) and an annual decline in eGFR_Scr-Scys_ of only − 0.6 mL/min/1.73 m^2^/year over two years. The analyses compared each of the two case groups with the non-cases. Given the small number of affected females exhibiting a rapid decline in kidney function (3 cases), formal analyses were restricted to males.

### Variables

Each participant completed a baseline questionnaire by face-to-face interview that included demographic data, current and previous occupations, lifestyle factors, medications, liquid intake, and dehydration symptoms [13, 14]. The season was defined as summer and winter. Work performed was classified as outdoor and indoor work. For this analysis, we used the eGFR and uNGAL levels, but in a sensitivity analysis the baseline questionnaire data (age, season, work performed, urinary albumin-creatinine ratio (UACR)) were included as part of the full model. UACR was categorized as a ratio ≥ 30 mg/g or < 30 mg/g [20].

### Statistical analysis

Descriptive analyses of continuous variables (age, serum creatinine, eGFR_Scr_, eGFR_Scr-Scys_, and uNGAL) stratified by case/non-case status were conducted, and are reported as the means (SD) or medians and interquartile ranges (IQRs). Categorical variables (season, work performed and UACR) are reported as frequencies and percentages.

For the identification of cases of MeN in the local setting, local measurements are required; therefore, the current analysis is based on the routine creatinine measurements performed after each visit. The agreement between the estimated GFR based on the Scr Jaffe assay conducted at the laboratory in Nicaragua and the eGFR_Scr_ from Oxford (gold-standard measure) was evaluated at three time points (baseline, 6 and 12 months) using a Bland-Altman plot.

For case-group 1, we first assessed whether the routine eGFR data derived from local creatinine measurements identified people with established kidney damage. Logistic regression models were also performed to compare case-group 1 with non-cases. Model 1 included eGFR_Scr_ at baseline; Model 2 included the Model 1 covariate plus uNGAL levels. Along with variables included in Models 1 and 2, subsequent models for non-cases and case-group 2 also included the 6- and/or 12-month eGFR with or without uNGAL levels.

For all models, receiver operating characteristic (ROC) curves were generated to calculate the area under the ROC curve (AUC). An AUC greater than 0.80 was considered a suitable discrimination performance for the model.

In order to check that the results are not confounded, we repeated the analysis using only the continuous eGFR, and uNGAL measurements with further adjustments for other variables such us age, season, work performed, and urinary albumin-creatinine ratio (UACR) (Table 1).

**Table 1:** Variables outlined in each of the models stratified by case-groups

Variables	Case-group 1		Case-group 2							
	*Model 1*	*Model 2*	*Model 3*	*Model 4*	*Model 5*	*Model 6*	*Model 7*	*Model 8*	*Model 9*	*Model 10*
Age	X	X	X	X	X	X	X	X	X	X
Season	X	X	X	X	X	X	X	X	X	X
Occupation (outdoor work)	X	X	X	X	X	X	X	X	X	X
UACR	X	X	X	X	X	X	X	X	X	X
UNGAL		X		X		X		X		X
eGFR_Scr_ at baseline	X	X	X	X	X	X	X	X	X	X
eGFR_Scr_ at 6 months					X	X			X	X
eGFR_Scr_ at 12 months							X	X	X	X

All statistical analyses were performed using Stata software, version 14 (Stata Corp.).

## Results

### Agreement between clinical measurements – Routine eGFR_Scr-Nicaragua_ compared to the gold-standard eGFR_Scr-Oxford_

In daily clinical practice, eGFR_Scr_ measurements are used, and we compared these values to gold-standard eGFR_Scr-Oxford_. The mean difference in baseline kidney function based on serum creatinine levels measured in Nicaragua compared to serum creatinine levels measured in Oxford was − 10.47 mL/min/1.73 m^2^ (95% CI -11.90 to − 9.03), suggesting that Nicaraguan kidney function was overestimated by local measurements. The limits of agreement ranged from − 37.24 to 16.30 mL/min/1.73 m^2^ with a variability of 3.7%, suggesting that local measurements varied. No evidence of the measurement error in eGFR_Scr_ was observed, with a poor correlation between the difference and the sums (*r* = − 0.06; *P* = 0.213) (Additional file 1: Figure S1A). The mean difference in eGFR_Scr-Nicaragua_ after 6 months was − 8.39 mL/min/1.73 m^2^ (95% CI -9.91 to − 6.88), with a range of 22.07 to 152.35 mL/min/1.73 m^2^. The limits of agreement were − 35.09 to 18.30 mL/min/1.73 m^2^ and the variability was 5.4% (Additional file 1: Figure S1B). Finally, the mean difference in eGFR_Scr-Nicaragua_ at the third study visit was + 3.44 mL/min/1.73 m^2^ (95% CI 1.76 to 5.11), with a range of 15.54 to 146.33 mL/min/1.73 m^2^. The limits of agreement were − 26.90 to 33.17 mL/min/1.73 m^2^ and the variability was 7.2%. A weak correlation between the difference and the sums was observed for both lab results (*r* = − 0.11; *P* = 0.042) (Additional file 1: Figure S1C).

### General characteristics stratified by kidney function trajectories

The mean age at baseline for the entire men population was 23.7 ± 3.8 years. The majority of males (74%) reported that they worked outdoors. Table 2 shows the routine local clinical kidney measurements at baseline stratified according to the observed renal outcome groups after 2 years of follow-up. At baseline, no differences were observed between controls and case-group 2. However, a subgroup of males had a high serum creatinine level and low eGFR_Scr_ at baseline, and were unaware of this condition at recruitment into the study (case-group 1). Case-group 1 displayed a high urinary uNGAL level at baseline. However, the percentages of males displaying a UACR ≥30 mg/g were 4.7% among participants with stable kidney function and 16% among participants with established renal dysfunction (case-group 1).

**Table 2:** Baseline characteristics of apparently healthy young men in northwest Nicaragua stratified by trajectories of a future decline in kidney function using gold standard measurements (CKDEPI Creat/Cyst eGFR) (*n* = 263)

Trajectories of the decline in kidney function	N	Age at baseline (Mean; SD)	Serum creatinine level at baseline‡ (Median; IQR)	eGFR_Scr_ at baseline‡ (Median; IQR)	eGFR_Scr-Scys_ at baseline† (Median; IQR)	Urinary NGAL level at baseline◊ (Median; IQR)	UACR ≥30 mg/dL at baseline◊ (n; %)	Outdoor work at baseline (n; %)
Stable kidney function (−0.6 mL/min/1.73 m^2^/year)	213	23.6 ± 3.89	0.76 (0.57–0.76)	131 (124–140)	118 (108–125)	5.04 (4.7–5.4)	10 (4.7)	151 (70.9)
Rapid decline in kidney function (− 18.2 mL/min/1.73 m^2^/year)	25	23.3 ± 3.65	0.66 (0.47–0.76)	132 (123–152)	117 (103–124)	5.20 (5.0–5.5)	0 (0)	24 (96.0)
Renal dysfunction (− 3.8 mL/min/1.73 m^2^/year)	25	25.4 ± 2.97	1.23 (1.14–1.52)	80 (62–91)	56 (49–68)	5.7 (5.5–5.7)	4 (16.0)	19 (76.0)
Total	263	23.7 ± 3.82	0.76 (0.66–0.85)	129 (121–139)	116 (102–125)	5.20 (4.9–5.7)	14 (5.3)	194 (73.8)

*‡Routine local test measurement. †Gold-standard outcome measured in Oxford 2 years later. ◊Urinary biomarkers of kidney injury*

### Model for predicting established renal dysfunction

**Fig. 1:**
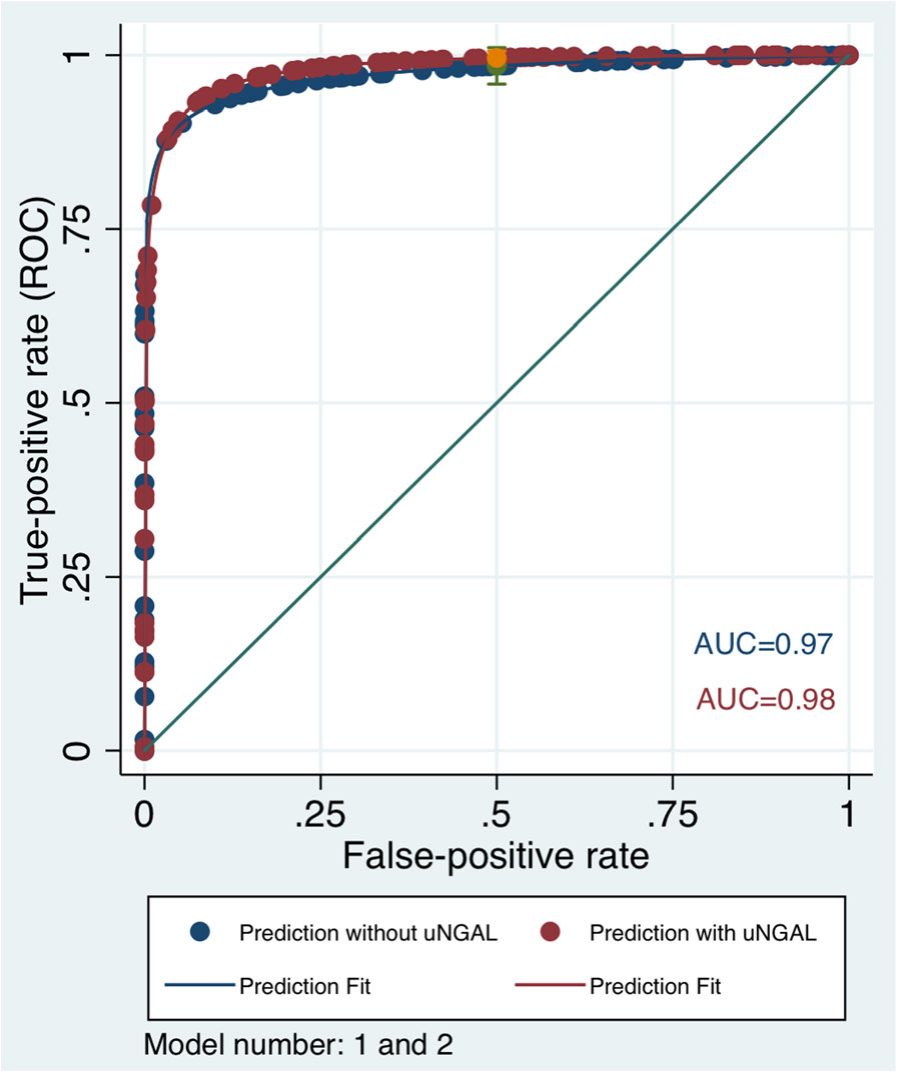
ROC curves for the model predicting stable kidney function versus established renal dysfunction using eGFR and uNGAL. The 95% confidence intervals for the ROC curves (0.5) are displayed

The prediction score comparing stable kidney function (non-cases) and established renal dysfunction (case-group 1) without uNGAL levels by using a single eGFR_Scr_ measurement was excellent (*P* < 0.001), showing an AUC of 0.97 (95% CI 0.93–1.00) (Additional file 1: Table S1; Model 1). The AUC for Model 2, where a single eGFR_Scr_ measurement and uNGAL levels did not improve the prediction compared with established predictors, was 0.98 (95% CI 0.95–1.00). The c statistic was the same for both models. (Fig. 1 and Additional file 1: Table S1).

### Model for predicting a rapid decline in kidney function

**Fig. 2:**
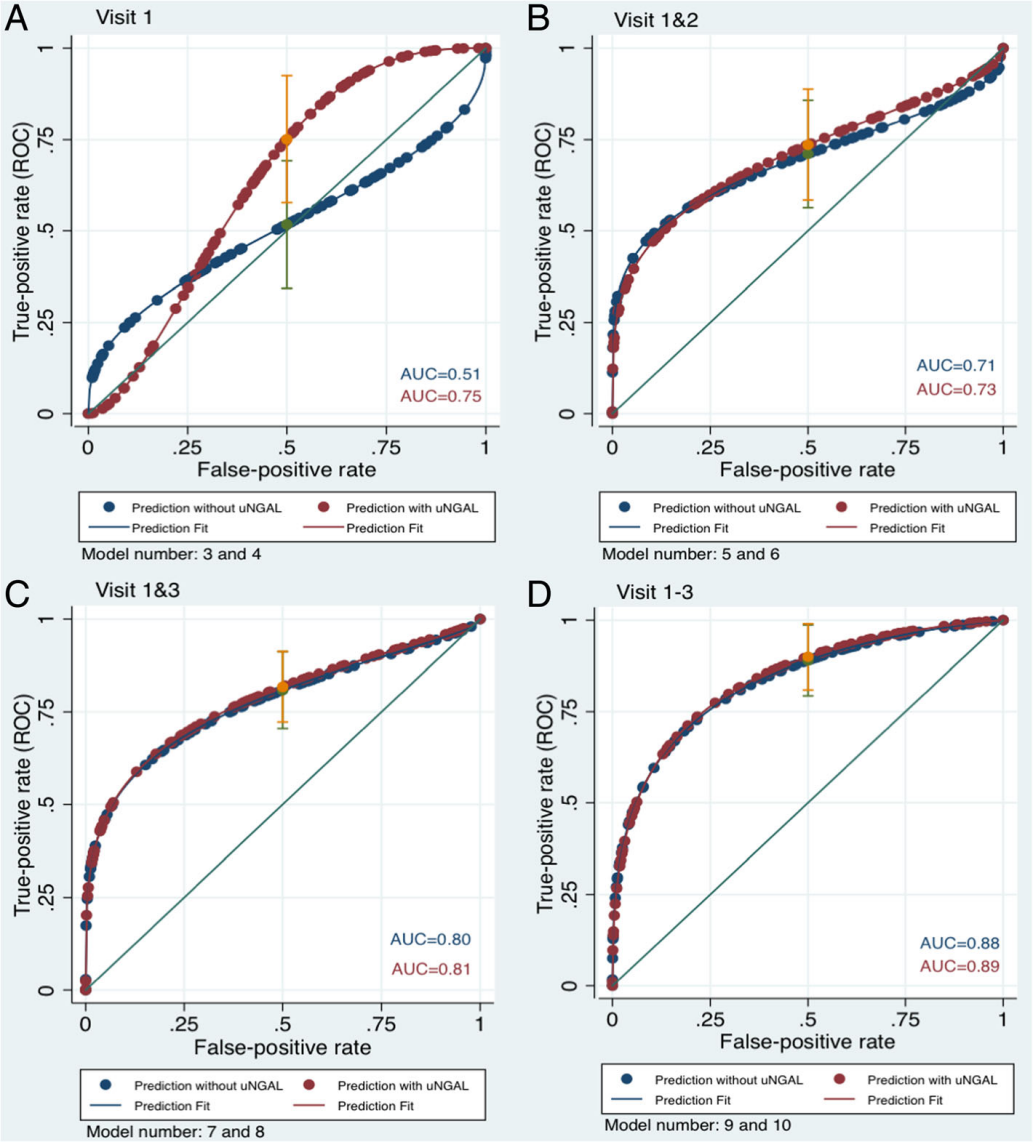
ROC curves for the model predicting stable kidney function versus a rapid decline in kidney function using eGFR and uNGAL. The 95% confidence intervals for ROC curves (0.5) are displayed. The blue label means: Area under the curve without uNGAL. The maroon label means: Area under the curve with uNGAL

The AUC just for the estimated glomerular filtration rate at baseline showed a poor discrimination to identify populations at risk of a rapid decline in kidney function (case-group 2). As shown in Fig. 2a, the discrimination power of the model at baseline was poor, with an AUC of 0.51 (95% CI 0.34–0.69), and when uNGAL was added to the logistic regression model, the AUC was 0.75 (95% CI 0.57–0.92). The c statistic for Model 4 with uNGAL levels was 0.24 higher, indicating a significant improvement compared with Model 3 that did not include uNGAL levels.

Figure 2b shows the results of the model without uNGAL levels, but with an additional measurement of eGFR_Scr_ at 6 months (Model 5). The discrimination power of the model was somewhat improved (compared to Model 3), with an AUC of 0.71 (95% CI 0.56–0.85). For the same variables with the addition of uNGAL levels (Model 6), the AUC was 0.73 (95% CI 0.58–0.88). The c statistic for model 6 (with uNGAL levels) was 0.02.

Figure 2c shows the results of the models including an eGFR_Scr_ recorded 12 months after the baseline measure (excluding the 6-month measurement; Model 7), with an AUC of 0.80 (95% CI 0.70–0.91). The addition of uNGAL levels (Model 8) to this model yielded an AUC of 0.81 (95% CI 0.72–0.91). The c statistic was almost indistinguishable between model 7 and model 8, suggesting that uNGAL levels do not additional predictive value to model 7.

The best prediction of a future rapid decline in kidney function (Fig. 2d) was achieved using 3 eGFR_Scr_ measurements: baseline, 6 months and 12 months (Model 9). This model yielded an AUC of 0.88 (95% CI 0.79–0.98) and an AUC of 0.89 (95% CI 0.80–0.99) when urinary uNGAL levels were incorporated into the model (Model 10). Again, the c statistics were similar between Models 9 and 10 (Fig. 2 and Additional file 1: Table S2).

Results of the sensitivity analysis using eGFR, uNGAL measurements plus baseline questionnaire data (age, season, work performed, and UACR) were not appreciably changed when compared to the main analysis (Additional file 1: Figures S2 and S3 and Additional file 1: Table S3 and S4).

## Discussion

Using a gold-standard measurement of decline in kidney function, we classified the participants into three categories: (i) established renal dysfunction (case-group 1); (ii) a rapid decline in kidney function (case-group 2); and (iii) stable kidney function (non-cases). We then compared each of the two case-groups with the non-cases. While it was straightforward to identify individuals with already established renal dysfunction at baseline using routine laboratory data together with uNGAL (AUC: 0.98; 95% CI: 0.95 to 1.00), a follow-up for at least one year with 6 monthly measurements was required to identify most people who will suffer from a future rapid decline in kidney function from normal participants (AUC: 0.89; 95% CI: 0.80 to 0.99 and a false positive rate of 0.5). We did not observe a difference in the ROC values in the final model with and without uNGAL levels and note that the confidence intervals nearly overlapped.

Good quality laboratory results are very important for an accurate diagnosis and clinical decision making [21–24]. The laboratory in Nicaragua is not accredited to ISO standards; however, as happens in routine clinical care, the machines are regularly recalibrated. Routine clinical care procedure do not standardly bank blood samples for several years to then retrospectively assess decline in kidney function. We showed that routine clinical care measurements displayed considerable time-dependent variability. The variability in the local Scr measurements increased the imprecision between the eGFR_Scr-Nicaragua_ and the gold-standard (eGFR_Scr-Oxford_) of 3.7% at baseline and 7.2% at the third study visit. However, for the eGFR determinations performed following the baseline visit, measurement error appeared to have decreased as the laboratory obtaining a better machine recalibration, maintenance and the implementation of external quality control standards. Based on these findings, health care providers and researchers must be aware of the challenges of using local serum creatinine measurements.

To date, many prediction models have been developed and validated for determining the progression of kidney disease to ESRD [25, 26]. Roy et al. developed a prediction model and showed how a novel biomarker (uNGAL) improves prediction of CKD progression in the CRIC study [27]. Their model included demographic data plus uNGAL levels, and the model without uNGAL levels showed a poor discrimination power (AUC = 0.69), whereas the model with uNGAL levels exhibited good discrimination (AUC = 0.82). Our model with uNGAL levels used to distinguish participants with renal dysfunction (case-group 1) did not add predictive value beyond the established predictors (AUC = 0.98) compared to the model without uNGAL levels (AUC = 0.97).

When predicting a future rapid decline in kidney function (case-group 2), the addition of uNGAL levels to the model that included a single eGFR_Scr_ measurement increased the AUC from 0.51 to 0.75 (Model 3 vs Model 4). However, in the models including three eGFR measurements (Model 9 vs Model 10) the two curves were almost indistinguishable (e.g., AUCs with and without uNGAL levels were 0.88 and 0.89 respectively for models 9 and 10). Thus, no discernible improvement in prediction was observed when uNGAL levels were added to a model containing more than one creatinine measurement recorded over time.

The key implication from our study for future cohort and/or intervention studies of CKDu is that the minimal follow-up period is one year, and 6 monthly measurements are required to distinguish the progressive loss of eGFR from stable kidney function. Any study with a shorter follow-up period less likely to detect significant progression among participants with normal function at baseline.

Our study has methodological strengths. First, this study is derived from a community-based longitudinal study of apparently healthy young adults in high-risk areas for CKDu. Second, the variables that were used to calculate the prediction score are easy to obtain at any health level (i.e., primary, secondary and tertiary health levels) and can be applied by doctors or any health professional.

Our study has some limitations that should be noted. External validation was not performed because this community-based cohort study is the first in the region, and no other comparable cohorts are available. Second, we were unable to assess the effect of the season in which eGFR measures are performed because almost all participants were recruited before the harvest season [14].

## Conclusions

In conclusion, established renal dysfunction was detected at a single time point using local measurements of eGFR and uNGAL. However, the detection of a rapid decline in kidney function over time requires at least 2 measurements, ideally at least twelve months apart. In addition, local routine clinical measurements of creatinine levels are affected by time-dependent measurement error.

## Additional file

Additional file 1: **Figure S1.** Bland-Altman plot of eGFR based on serum creatinine levels measured in Nicaragua and serum creatinine levels measured in London. **Figure S2*.*** ROC curves for the model predicting stable kidney function versus established renal dysfunction. The 95% confidence intervals for the ROC curves (0.5) are displayed. **Figure S3*.*** ROC curves for the model predicting stable kidney function versus a rapid decline in kidney function. The 95% confidence intervals for ROC curves (0.5) are displayed. **Table S1*.*** Multivariate adjusted logistic regression analysis for eGFR and uNGAL with established renal dysfunction at baseline among apparently healthy young males. **Table S2.** Multivariate adjusted logistic regression analysis for eGFR and uNGAL with a rapid decline in kidney function at baseline among apparently healthy young males. **Table S3.** Multivariate adjusted logistic regression analysis of factors associated with established renal dysfunction at baseline among apparently healthy young males. **Table S4*.*** Multivariate adjusted logistic regression analysis for factors associated with a rapid decline in kidney function at baseline among apparently healthy young males. (PDF 600 kb)

## Abbreviations

AUC: Area under the receiver operating characteristic curve
CI: Confidence interval
CISTA: Centro de Investigación en Salud, Trabajo y Ambiente
CKD: Chronic kidney disease
CKD-EPI: Chronic Kidney Disease Epidemiology Collaboration
CKDu: Chronic kidney disease of unknown aetiology
CRIC: Chronic renal insufficiency cohort
eGFR: Estimated glomerular filtration rate
ESRD: End-stage renal disease
GMM: Growth mixture model
IDMS: Isotope dilution mass spectrometry
IQR: Interquartile range
MeN: Mesoamerican nephropathy
NAG: N-acetyl-β-D-glucosaminidase
ROC: Receiver operating characteristic
Scr: Serum creatinine
Scys: Serum cystatin C
SD: Standard deviation
UACR: Urinary albumin-creatinine ratio
UK: United Kingdom
uNGAL: Urinary neutrophil gelatinase-associated lipocalin
