# Supplementary material for: Identification of young adults at risk of an accelerated loss of kidney function in an area affected by Mesoamerican nephropathy
Source: BMC Nephrol. 2019 Jan 16;20:21. doi: 10.1186/s12882-018-1193-x (PMC6335797; doi:10.1186/s12882-018-1193-x)

Identification of young adults at risk of accelerated kidney function loss in an area affected by Mesoamerican nephropathy

Marvin Gonzalez-Quiroz[1,2,3], Evangelia-Theano Smpokou[3], Neil Pearce[2], Ben Caplin[3], Dorothea Nitsch[2]**

[1]Research Centre on Health, Work and Environment (CISTA), National Autonomous University of Nicaragua at León (UNAN-León), León, Nicaragua. [2]Department of Non-Communicable Disease Epidemiology, London School of Hygiene and Tropical Medicine, London, UK. [3]Centre for Nephrology, University College London, London, UK.

Supplementary Material

Figures:

Figure S1: Bland-Altman for eGFR based on serum creatinine levels measured in Nicaragua and serum creatinine levels measured in London.

Figure S2: ROC curves for the model predicting stable kidney function versus established renal dysfunction. The 95% confidence intervals for the ROC curves (0.5) are displayed.

Figure S3: ROC curves for the model predicting stable kidney function versus a rapid decline in kidney function. The 95% confidence intervals for ROC curves (0.5) are displayed.

Tables:

Table S1: Multivariate adjusted logistic regression analysis for eGFR and uNGAL associated with established renal dysfunction at baseline among apparently healthy young males

Table S2: Multivariate adjusted logistic regression analysis for eGFR and uNGAL associated with a rapid decline in kidney function at baseline among apparently healthy young males

Table S3: Multivariate adjusted logistic regression analysis of factors associated with established renal dysfunction at baseline among apparently healthy young males

Table S4: Multivariate adjusted logistic regression analysis for factors associated with a rapid decline in kidney function at baseline among apparently healthy young males

Figure S1: Bland-Altman for eGFR based on serum creatinine levels measured in Nicaragua and serum creatinine levels measured in London.

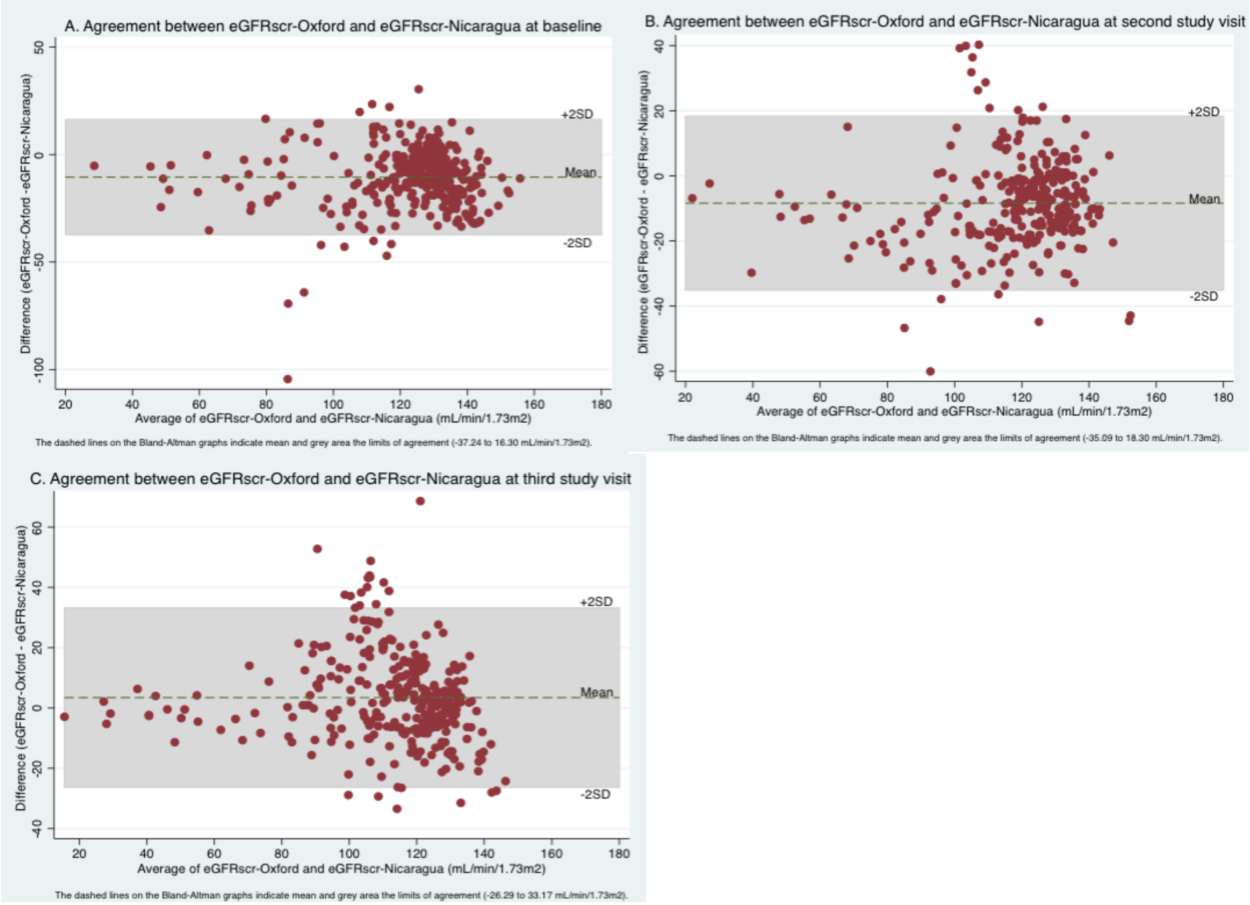

Table S1: Multivariate adjusted logistic regression analysis for eGFR and uNGAL associated with established renal dysfunction at baseline among apparently healthy young males

Factors	Model 1 (n=80)				Model 2 (n=80)			
	Without uNGAL levels				With uNGAL levels			
	Coefficient	SE	95% CI	P-value	Coefficient	SE	95% CI	P-value
$eGFR_{Scr}$ at baseline	-0.14	0.03	-0.22 to -0.07	<0.001	-0.15	0.04	-0.24 to -0.06	<0.001
Urinary NGAL	--	--	--	--	3.16	1.44	0.34 to 5.99	0.028

Abbreviations: SE: standard error, uNGAL: urinary neutrophil gelatinase-associated lipocalin, $eGFR_{Scr}$: estimated glomerular filtration rate based on locally measured creatinine levels.

Table S2: Multivariate adjusted logistic regression analysis for eGFR and uNGAL associated with a rapid decline in kidney function at baseline among apparently healthy young males

Factors	Figure 2A								Figure 2B			
	Model 3 without uNGAL levels (n=79)				Model 4 with uNGAL levels (n=79)				Model 5 without uNGAL levels (n=68)			
	Coefficient	SE	95% CI	P-value	Coefficient	SE	95% CI	P-value	Coefficient	SE	95% CI	P-value
$eGFR_{Scr}$ at baseline	0.003	0.01	-0.02 to 0.03	0.808	0.004	0.01	-0.02 to 0.03	0.798	-0.01	0.01	-0.04 to 0.02	0.507
Urinary NGAL	-	-	-	-	0.81	0.45	-0.06 to 1.70	0.070	-	-	-	-
$eGFR_{Scr}$ at second study visit	-	-	-	-	-	-	-	-	-0.06	0.02	-0.10 to -0.01	0.015
$eGFR_{Scr}$ at third study visit	-	-	-	-	-	-	-	-	-	-	-	-

Abbreviations: SE: standard error, uNGAL: urinary neutrophil gelatinase-associated lipocalin, $eGFR_{Scr}$: estimated glomerular filtration rate based on locally measured creatinine levels.

Continued: Table S2: Multivariate adjusted logistic regression analysis for eGFR and uNGAL associated with a rapid decline in kidney function at baseline among apparently healthy young males

Factors	Figure 2B				Figure 2C							
	Model 6 with uNGAL levels (n=68)				Model 7 without uNGAL levels (n=73)				Model 8 with uNGAL levels (n=73)			
	Coefficient	SE	95% CI	P-value	Coefficient	SE	95% CI	P-value	Coefficient	SE	95% CI	P-value
$eGFR_{Scr}$ at baseline	-0.01	0.01	-0.04 to 0.02	0.501	0.01	0.01	-0.01 to 0.05	0.328	0.01	0.01	-0.01 to 0.05	0.330
Urinary NGAL	0.54	0.95	-0.57 to 1.65	0.310	-	-	-	-	0.56	0.56	-0.54 to 1.67	0.319
$eGFR_{Scr}$ at second study visit	-0.05	0.02	-0.10 to -0.006	0.025	-	-	-	-	-	-	-	-
$eGFR_{Scr}$ at third study visit	-	-	-	-	-0.06	0.01	-0.09 to -0.02	<0.001	-0.05	0.01	-0.09 to -0.02	0.001

Abbreviations: SE: standard error, uNGAL: urinary neutrophil gelatinase-associated lipocalin, $eGFR_{Scr}$: estimated glomerular filtration rate based on locally measured creatinine levels.

Continued: Table S2: Multivariate adjusted logistic regression analysis for eGFR and uNGAL associated with a rapid decline in kidney function at baseline among apparently healthy young males

Factors	Figure 2D							
	Model 9 without uNGAL levels (n=67)				Model 10 with uNGAL levels (n=67)			
	Coefficient	SE	95% CI	P-value	Coefficient	SE	95% CI	P-value
$eGFR_{Scr}$ at baseline	-0.004	0.01	-0.04 to 0.03	0.812	-0.004	0.01	-0.04 to 0.03	0.798
$eGFR_{Scr}$ at second study visit	-0.02	0.02	-0.07 to 0.02	0.404	-0.01	0.02	-0.06 to 0.03	0.485
$eGFR_{Scr}$ at third study visit	-0.06	0.02	-0.10 to -0.02	0.002	-0.06	0.02	-0.10 to -0.02	0.002
Urinary NGAL	-	-	-	-	0.44	0.66	-0.86 to 1.74	0.508

Abbreviations: SE: standard error, uNGAL: urinary neutrophil gelatinase-associated lipocalin, $eGFR_{Scr}$: estimated glomerular filtration rate based on locally measured creatinine levels.

Table S3: Multivariate adjusted logistic regression analysis of factors associated with established renal dysfunction at baseline among apparently healthy young males

Factors	Model 1 (n=74)				Model 2 (n=74)			
	Without uNGAL levels				With uNGAL levels			
	Coefficient	SE	95% CI	P-value	Coefficient	SE	95% CI	P-value
Age	0.14	0.22	-0.29 to 0.57	0.525	0.12	0.22	-0.30 to 0.56	0.563
Season								
Summer	0.0	--	--	--	0	--	--	--
Winter	1.00							
Outdoor work								
Yes	7.14	4.14	-0.97 to 15.26	0.084	7.57	5.42	-3.05 to 18.20	0.162
No	1.00				1.00			
Urinary ACR								
≥30 mg/g	0	--	--	--	0	--	--	--
<30 mg/g	1.00				1.00			
Urinary NGAL	--	--	--	--	3.31	1.87	0.35 to 6.99	0.077
$eGFR_{Scr}$ at baseline	-0.26	0.10	-0.47 to -0.05	0.014	-0.27	0.13	-0.54 to -0.005	0.045

Abbreviations: SE: standard error, uNGAL: urinary neutrophil gelatinase-associated lipocalin, UACR: urinary albumin creatinine ratio, $eGFR_{Scr}$: estimated glomerular filtration rate based on locally measured creatinine levels.

Table S4: Multivariate adjusted logistic regression analysis of factors associated with a rapid decline in kidney function at baseline among apparently healthy young males

Factors	Figure 2A								Figure 2B			
	Model 3 without uNGAL levels (n=78)				Model 4 with uNGAL levels (n=78)				Model 5 without uNGAL levels (n=67)			
	Coefficient	SE	95% CI	P-value	Coefficient	SE	95% CI	P-value	Coefficient	SE	95% CI	P-value
Age	-0.06	0.06	-0.19 to 0.06	0.354	-0.06	0.06	-0.20 to 0.06	0.327	-0.11	0.09	-0.29 to 0.05	0.187
Season												
Summer	0	--	--	--	0	--	--	--	0	--	--	--
Winter	1.00				1.00				1.00			
Outdoor work												
Yes	1.59	1.08	-0.54 to 3.72	0.144	1.77	1.10	-0.39 to 3.94	0.110	1.42	1.12	-0.78 to 3.63	0.206
No	1.00				1.00				1.00			
Urinary ACR												
≥30 mg/g	0	--	--	--	0	--	--	--	0	--	--	--
<30 mg/g	1.00				1.00				1.00			
Urinary NGAL	-	-	-	-	0.97	0.49	-0.0008 to 1.94	0.050	-	-	-	-
$eGFR_{Scr}$ at baseline	-0.004	0.01	-0.03 to 0.02	0.789	-0.004	0.01	-0.03 to 0.02	0.794	-0.02	0.02	-0.06 to 0.01	0.237
$eGFR_{Scr}$ at second study visit	-	-	-	-	-	-	-	-	-0.06	0.02	-0.11 to -0.01	0.018
$eGFR_{Scr}$ at third study visit	-	-	-	-	-	-	-	-	-	-	-	-

Abbreviations: SE: standard error, UACR: urinary albumin creatinine ratio, uNGAL: urinary neutrophil gelatinase-associated lipocalin, $eGFR_{Scr}$: estimated glomerular filtration rate based on locally measured creatinine levels.

Continued: Table S4: Multivariate adjusted logistic regression analysis of factors associated with a rapid decline in kidney function at baseline among apparently healthy young males

Factors	Figure 2B				Figure 2C							
	Model 6 with uNGAL levels (n=67)				Model 7 without uNGAL levels (n=72)				Model 8 with uNGAL levels (n=72)			
	Coefficient	SE	95% CI	P-value	Coefficient	SE	95% CI	P-value	Coefficient	SE	95% CI	P-value
Age	-0.11	0.08	-0.29 to 0.06	0.202	-0.18	0.10	-0.38 to 0.01	0.068	-0.18	0.09	-0.37 to 0.01	0.066
Season												
Summer	0	--	--	--	0	--	--	--	0	--	--	--
Winter	1.00				1.00				1.00			
Outdoor work												
Yes	1.48	1.13	-0.73 to 3.71	0.190	3.12	2.01	-0.81 to 7.07	0.120	3.35	2.14	-0.84 to 7.55	0.117
No	1.00				1.00				1.00			
Urinary ACR												
≥30 mg/g	0	--	--	--	0	--	--	--	0	--	--	--
<30 mg/g	1.00				1.00				1.00			
Urinary NGAL	0.63	0.62	-0.58 to 1.84	0.310	-	-	-	-	0.76	0.63	-0.48 to 2.01	0.229
$eGFR_{Scr}$ at baseline	-0.02	0.02	-0.06 to 0.01	0.243	-0.0001	0.01	-0.03 to 0.03	0.996	0.0006	0.01	-0.03 to 0.03	0.972
$eGFR_{Scr}$ at second study visit	-0.05	0.02	-0.10 to -0.004	0.034	-	-	-	-	-	-	-	-
$eGFR_{Scr}$ at third study visit	-	-	-	-	-0.07	0.02	-0.11 to -0.03	<0.001	-0.07	0.02	-0.11 to -0.03	<0.001

Abbreviations: SE: standard error, uNGAL: urinary neutrophil gelatinase-associated lipocalin, UACR: urinary albumin creatinine ratio, $eGFR_{Scr}$: estimated glomerular filtration rate based on locally measured creatinine levels.

Continued: Table S4: Multivariate adjusted logistic regression analysis of factors associated with a rapid decline in kidney function at baseline among apparently healthy young males

Factors	Figure 2D							
	Model 9 without uNGAL levels (n=66)				Model 10 with uNGAL levels (n=66)			
	Coefficient	SE	95% CI	P-value	Coefficient	SE	95% CI	P-value
Age	-0.25	0.12	-0.50 to 0.002	0.053	-0.23	0.12	-0.49 to 0.01	0.065
Season								
Summer	0	--	--	--	0	--	--	--
Winter	1.00				1.00			
Outdoor work								
Yes	3.40	2.24	-1.00 to 7.81	0.130	3.47	2.29	-1.02 to 7.97	0.130
No	1.00				1.00			
Urinary ACR								
≥30 mg/g	0	--	--	--	0	--	--	--
<30 mg/g	1.00				1.00			
Urinary NGAL	-	-	-	-	0.42	0.73	-1.01 to 1.86	0.561
$eGFR_{Scr}$ at baseline	-0.02	0.02	-0.07 to 0.01	0.239	-0.02	0.02	-0.06 to 0.01	0.254
$eGFR_{Scr}$ at second study visit	-0.03	0.02	-0.08 to 0.02	0.269	-0.02	0.02	-0.08 to 0.03	0.360
$eGFR_{Scr}$ at third study visit	-0.08	0.02	-0.12 to -0.03	0.001	-0.07	0.02	-0.12 to -0.03	0.001

Abbreviations: SE: standard error, uNGAL: urinary neutrophil gelatinase-associated lipocalin, UACR: urinary albumin creatinine ratio, $eGFR_{Scr}$: estimated glomerular filtration rate based on locally measured creatinine levels.

Figure S2: ROC curves for the model predicting stable kidney function versus established renal dysfunction. The 95% confidence intervals for the ROC curves (0.5) are displayed.

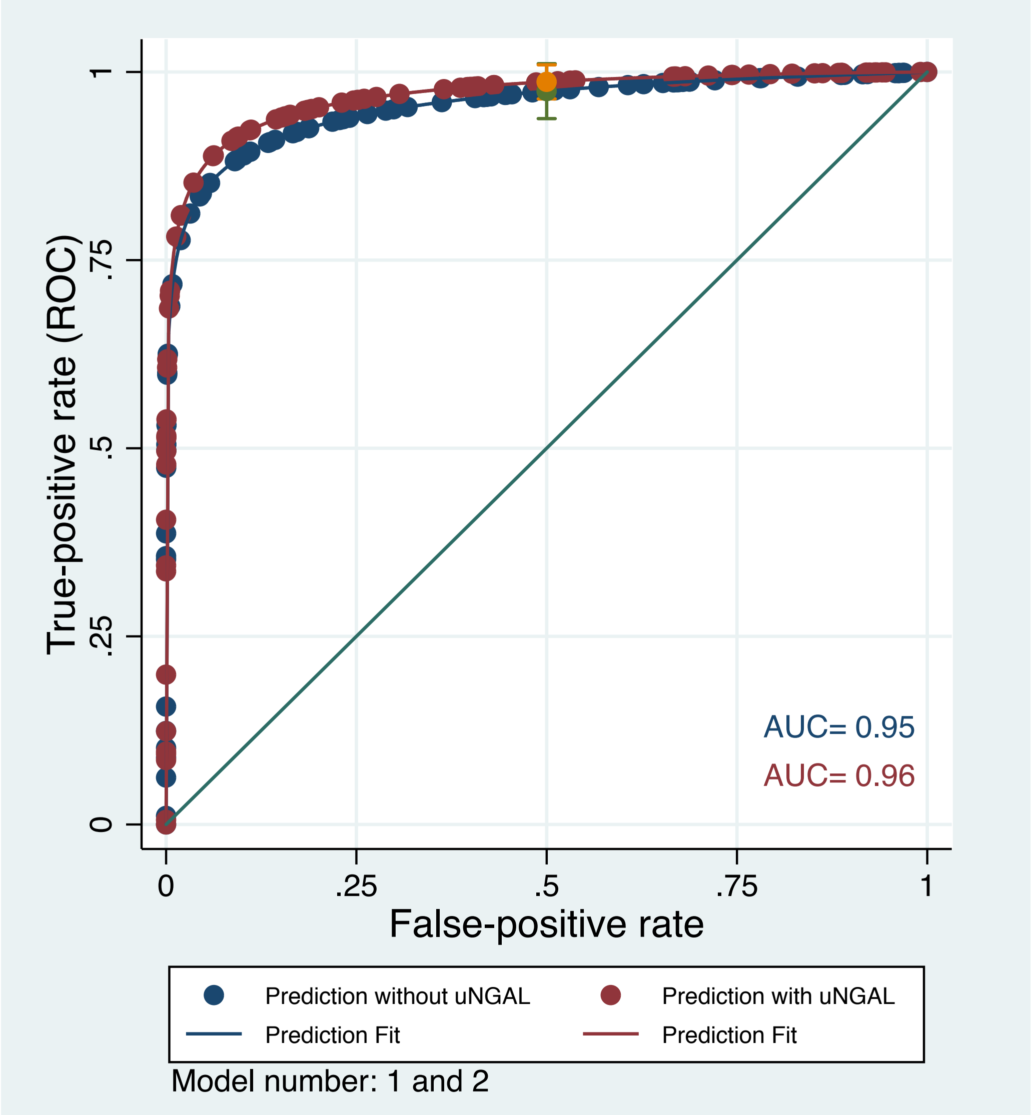

Figure S3: ROC curves for the model predicting stable kidney function versus a rapid decline in kidney function. The 95% confidence intervals for ROC curves (0.5) are displayed.

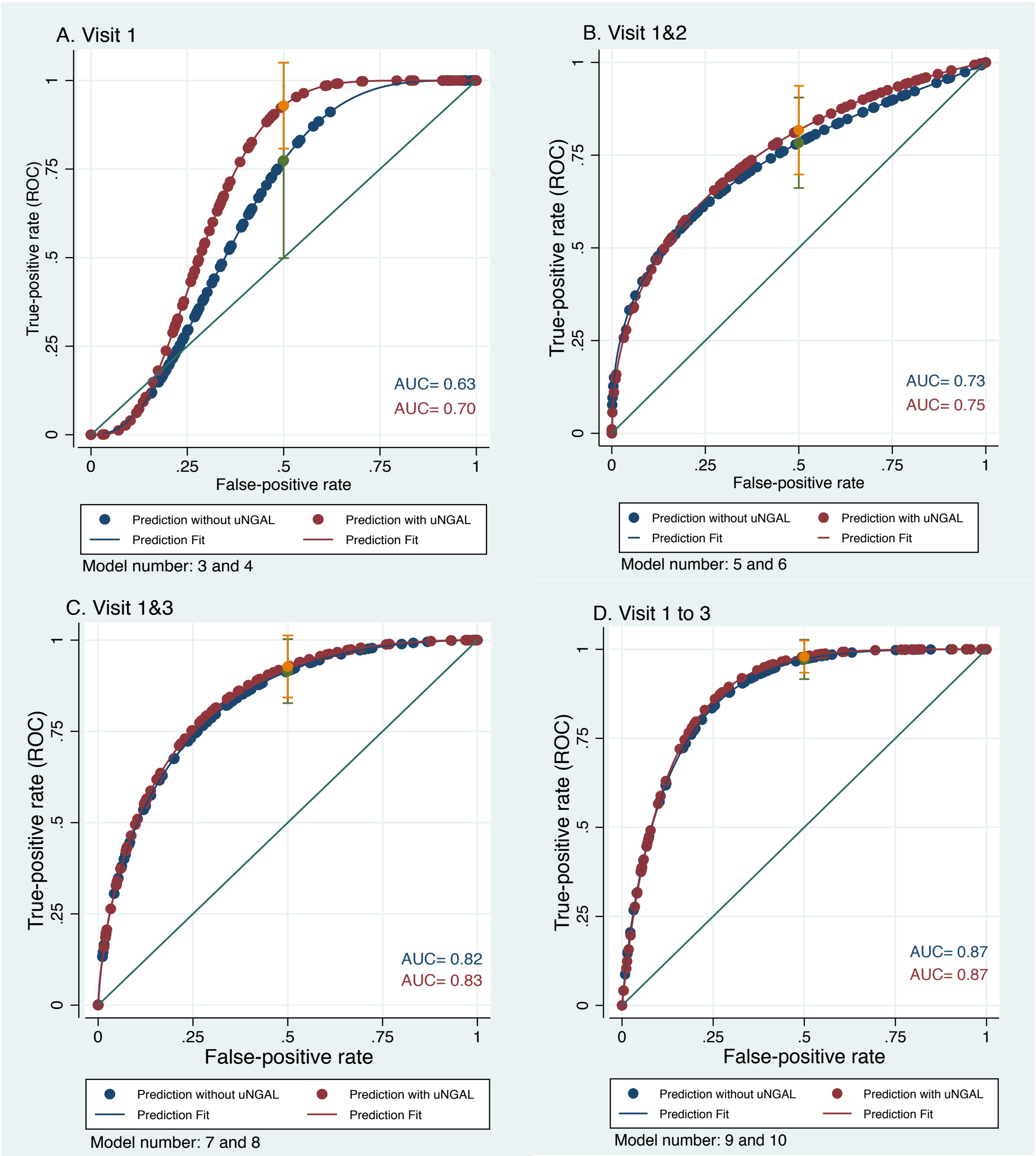

Supplement: Supplementary file 1 — Figure S1. Bland-Altman plot of eGFR based on serum creatinine levels measured in Nicaragua and serum creatinine levels measured in London. Figure S2. ROC curves for the model predicting stable kidney function versus established renal dysfunction. The 95% confidence intervals for the ROC curves (0.5) are displayed. Figure S3. ROC curves for the model predicting stable kidney function versus a rapid decline in kidney function. The 95% confidence intervals for ROC curves (0.5) are displayed. Table S1. Multivariate adjusted logistic regression analysis for eGFR and uNGAL with established renal dysfunction at baseline among apparently healthy young males. Table S2. Multivariate adjusted logistic regression analysis for eGFR and uNGAL with a rapid decline in kidney function at baseline among apparently healthy young males. Table S3. Multivariate adjusted logistic regression analysis of factors associated with established renal dysfunction at baseline among apparently healthy young males. Table S4. Multivariate adjusted logistic regression analysis for factors associated with a rapid decline in kidney function at baseline among apparently healthy young males. (PDF 600 kb) [file 12882_2018_1193_MOESM1_ESM.pdf]
